# Changing seroprevalence of hepatitis C virus infection among HIV-positive patients in Taiwan

**DOI:** 10.1371/journal.pone.0194149

**Published:** 2018-03-16

**Authors:** Chia-Wen Li, Chia-Jui Yang, Hsin-Yun Sun, Mao-Song Tsai, Shih-Ping Lin, Te-Yu Lin, Chien-Yu Cheng, Yi-Chien Lee, Yu-Shan Huang, Chun-Eng Liu, Yuan-Ti Lee, Hung-Jen Tang, Ning-Chi Wang, Shu-Hsing Cheng, Wen-Chien Ko, Chien-Ching Hung, for the Taiwan HIV Study Group

**Affiliations:** 1 Department of Internal Medicine, National Cheng Kung University Hospital, College of Medicine, National Cheng Kung University, Tainan, Taiwan; 2 Department of Internal Medicine, Far Eastern Memorial Hospital, New Taipei City, Taiwan; 3 School of Medicine, National Yang-Ming University, Taipei, Taiwan; 4 Department of Internal Medicine, National Taiwan University Hospital and National Taiwan University College of Medicine, Taipei, Taiwan; 5 Department of Internal Medicine, Catholic Fu-Jen Medical College, New Taipei City, Taiwan; 6 Department of Internal Medicine, Taichung Veterans General Hospital, Taichung, Taiwan; 7 Department of Internal Medicine, Tri-Service General Hospital and National Defense Medical Center, Taipei, Taiwan; 8 Department of Infectious Diseases, Taoyuan General Hospital, Ministry of Health and Welfare, Taoyuan, Taiwan; 9 School of Public Health, National Yang-Ming University, Taipei, Taiwan; 10 Department of Internal Medicine, Ditmanson Medical Foundation Chia-Yi Christian Hospital, Chia-Yi, Taiwan; 11 Department of Internal Medicine, National Taiwan University Hospital Hsin-Chu Branch, Hsin-Chu, Taiwan; 12 Department of Internal Medicine, Changhua Christian Hospital, Changhua, Taiwan; 13 School of Medicine, Chung Shan Medical University, Taichung, Taiwan; 14 Department of Internal Medicine, Chung Shan Medical University Hospital, Taichung, Taiwan; 15 Department of Internal Medicine, Chi Mei Medical Center, Tainan, Taiwan; 16 Department of Health and Nutrition, Chia Nan University of Pharmacy and Sciences, Tainan, Taiwan; 17 School of Public Health, Taipei Medical University, Taipei, Taiwan; 18 Department of Parasitology, National Taiwan University College of Medicine, Taipei, Taiwan; 19 Department of Medical Research, China Medical University Hospital, Taichung, Taiwan; 20 China Medical University, Taichung, Taiwan; Centers for Disease Control and Prevention, UNITED STATES

## Abstract

**Objective:**

The study aimed to describe the evolution of the seroprevalence of hepatitis C virus (HCV) among human immunodeficiency virus (HIV)-positive patients included in two cohorts in Taiwan.

**Methods:**

We retrospectively collected the information on demographic and clinical characteristics of 4,025 and 3,856 HIV-positive Taiwanese, who were aged 18 years or older at designated hospitals around Taiwan in 2004–2007, when an outbreak of HIV infection was occurring, and 2012–2016, when the outbreak was controlled with the implementation of harm reduction program, respectively. Comparisons of HCV seropositivity were made among different age and risk groups for HIV transmission between these two cohorts.

**Results:**

The overall HCV seroprevalence of the 2004–2007 cohort and 2012–2016 cohort was 43.4% (1,288/2,974) and 18.6% (707/3,793), respectively (*P*<0.001). The HCV seroprevalence among injecting drug users (IDUs), though decreasing, was constantly high across the two cohorts, 96.4% and 94.0% (*P* = 0.02), respectively, and all age groups. In contrast, the corresponding figures among men who have sex with men (MSM) and heterosexuals in the two cohorts were 5.9% *vs*. 3.5% (*P* = 0.002) and 9.4% *vs*. 10.9% (*P* = 0.59), respectively. Among sexually transmitted HIV-positive patients, HCV seropositivity was significantly correlated with age (adjusted odds ratio [aOR], per 1-year increase, 1.03; 95% confidence interval [CI], 1.02–1.05) and a rapid plasma reagin (RPR) titer ≥1:8 (aOR, 1.58; 95% CI, 1.03–2.43) in a multivariate analysis including age, gender, route for HIV transmission, baseline CD4 count and plasma HIV RNA load, the presence of hepatitis B surface antigen, and an RPR titer ≥1:8. Compared with heterosexuals, the aOR for HCV seropositivity among MSM was 0.47 (95% CI, 0.31–0.72).

**Conclusions:**

HCV seroprevalence among HIV-positive patients in Taiwan decreased with time, probably related to the inclusion of younger adults and more non-IDUs, and remained high among IDUs. HCV seropositivity was associated with age and an RPR titer ≥1:8 among patients who acquired HIV through sexual contact.

## Introduction

Hepatitis C virus (HCV) is a major cause of chronic liver disease, cirrhosis, and hepatocellular carcinoma [1,2]. Estimates of global prevalence of HCV infection range from 1.6% to 2.8%, corresponding to 80 to 185 million people [3–5]. In the past, HCV was mostly described as being acquired from transfusion of unscreened blood products, injecting drug use, accidental needle sticks, unsterile needle use during the medical procedures, and tattooing [6–8]. However, an increasing number of cases of sexually acquired HCV infection have been reported over the past two decades [9]; the factors identified to be associated with those incident HCV infections include male gender, an older age, infection with hepatitis B virus (HBV) and other hepatitis virus, substance use, sexually transmitted infections, and HIV infection [10].

Because HIV and HCV share the same transmission routes, the occurrence of co-infection is not uncommon. Among the HIV-positive individuals, it is estimated 20–30% are co-infected with HCV, and the disease progression among patients with HIV/HCV co-infection are more prominent compared with patients with HIV or HCV mono-infection [11]. The prevalence of HIV/HCV co-infection varies among the different regions and risk groups studied [12]. In China, the seroprevalence of HCV infection among HIV-positive injecting drug users (IDUs) ranged from 60% to 90%, with a pooled seroprevalence of 83%, while that among HIV-positive men who have sex with men (MSM) ranged from 0% to 24.2%, with a pooled seroprevalence of 4% [13]. In Southeast Asia, the seroprevalence of HCV co-infection among HIV-positive patients was estimated to be 2.9% to 80.8%, with significant regional differences [13].

While parenteral route is an efficient route for HCV transmission, recent emergence of sexually acquired HCV infections, particularly among HIV-positive MSM, has raised great concerns [9,14]. Previous studies have suggested that HIV-positive individuals with higher CD4 counts experienced more sexually transmitted infections after they returned to a healthier status after initiation of combination antiretroviral therapy (cART). Improved survival and serosorting among the HIV-positive individuals has been proposed to be related to these outbreaks of sexually transmitted HCV infections among MSM [15,16].

Few reports have investigated the changes of HCV seroprevalence among the HIV-positive individuals in the era of cART and harm reduction program for IDUs. In this multicenter study, we aimed to describe the seroepidemiology of HCV infection and to examine the evolution of HCV seroprevalence among HIV-positive Taiwanese in two study periods.

## Materials and methods

### Study setting and populations

In Taiwan, the total number of HIV-positive patients reported to Taiwan Centers for Disease Control (CDC) was 34,479 as of 31 December 2016 since the first case of HIV infection diagnosed in 1984, with an estimated HIV prevalence of 126.4 per 100,000 populations. Before 2004, the majority of HIV infections occurred through sexual transmission, with MSM accounting for the largest proportion (48.2%), followed by heterosexuals (39.9%) [17]. However, an outbreak of HIV infection occurred among IDUs between 2003 and 2008; IDUs accounted for two-thirds of all cases of newly diagnosed HIV infection in 2005 and 2006. The outbreak of HIV infection was controlled with the rapid and sustained implementation of harm reduction program consisting of needle exchange and methadone maintenance programs in 2005 [18]. During the outbreak of HIV infection among IDUs, up to 96% of HIV-positive IDUs were co-infected with HCV [19]. Afterwards, sexual contact remained the leading mode of transmission for HIV infection, accounting for at least 89.9% of the reported cases of HIV infection in 2016 [17]. HIV care, including cART and monitoring of plasma HIV RNA load (PVL) and CD4 count, is provided free-of-charge at designated hospitals around Taiwan [20].

To understand the evolution of HCV seroprevalence among the HIV-positive individuals, two retrospective cohort studies were conducted at major designated hospitals in Taiwan. The methods and results of the first cohort that included 4,025 HIV-positive adults (aged ≥18 years) between 2004 and 2007 have been reported previously (2004–2007 cohort) [21]. The second cohort included the adults initiating cART between June 2012 and May 2016 (2012–2016 cohort). A similar case record form was used to collect their demographic and clinical characteristics including birth date, gender, route of HIV transmission, serological data of viral hepatitis and syphilis, CD4 lymphocyte count, and PVL at baseline and during follow-up. The study was approved by the Research Ethics Committee (National Taiwan University Hospital [201003112R] and Far Eastern Memorial Hospital [105040-F]), Medical Ethics and Institutional Review Board of Taoyuan General Hospital [TYGH103011], and Institutional Review Boards (Tri-Service General Hospital [1-105-05-057] National Taiwan University Hospital Hsin-Chu Branch [105-017-F], Taichung Veterans General Hospital [CF16114B], Chung Shan Medical University Hospital [CS14034], Changhua Christian Hospital [160408], Chia-Yi Christian Hospital [105034], National Cheng Kung University Hospital [B-BR-105-038], and Chi Mei Medical Center [10505–002]). The informed consent was waived.

### Laboratory examinations

HIV infection was diagnosed by western blot test or detection of HIV viremia. Determinations of CD4 lymphocyte count and PVL, anti-HCV antibody, hepatitis B surface antigen (HBsAg) and anti-HBs antibody, anti-hepatitis A virus (HAV) antibody (HAV IgG), and the rapid plasma reagin (RPR) titer for syphilis were performed with the use of certified commercial kits at each participating hospital, and the results were collected locally and then pooled and analyzed.

### Statistical analysis

All statistical analyses were performed with the use of SPSS software version 21.0 (SPSS Inc., Chicago, IL, USA). Categorical variables, expressed as numbers and percentages, were compared using chi-squared test or Fisher’s exact test. Continuous variables were expressed as means ± standard deviation (SDs) and were compared with Mann-Whitney U-test. A multiple logistic regression model was built to identify independent variables associated with anti-HCV seropositivity. All tests were two tailed and a *P* value of <0.05 was considered significant.

## Results

In the 2004–2007 cohort, a total of 4,025 patients were included for analysis, with male predominance (n = 3,710, 92.2%) and a mean age of 38.6 years; among them, 1,478 (36.7%) patients acquired HIV through injecting drug use and 1,271 (31.6%) were MSM (Table 1) [21]. In contrast, in the 2012–2016 cohort that included 3,856 HIV-positive patients, 625 (16.2%) were IDUs and 2,948 (76.5%) MSM (Table 1). The mean CD4 lymphocyte count was 309.2 cells/μL (SD, 249 cells/μL) in the 2004–2007 cohort and 282 cells/μL (SD, 189 cells/μL) in the 2012–2016 cohort (*P*<0.001). The late presenters, defined as CD4 ≤200 cells/μL at the first visit, composed of 37.0% and 34.1% of the patients in the 2004–2007 cohort and 2012–2016 cohort, respectively (*P* = 0.011). The mean PVL of the former cohort was significantly lower than that in the later cohort (4.41 vs 4.73 log_10_ copies/mL, *P*<0.001).

**Table 1:** Comparisons of demographic and clinical characteristics between the two cohorts.

Variables	2004–2007 cohort	2012–2016 cohort	*P* value
	n = 4,025	n = 3,856	
Male gender, n (%)	3,710 (92.0)	3,675 (95.3)	<0.001
Mean age (SD, range), years	38.6 (11.1, 18–95)	33.1 (9.5, 18–84)	<0.001
Year of birth			<0.001
before 1950	266 (6.6)	22 (0.6)	
1950–1959	449 (11.2)	102 (2.6)	
1960–1969	1,178 (29.3)	387 (10)	
1970–1979	1,595 (39.6)	897 (23.3)	
1980–1989	537 (13.3)	1,762 (45.7)	
1990–2000	0	686 (17.8)	
Transmission risk group			<0.001
Men who have sex with men	1,271 (31.6)	2,948 (76.5)	
Heterosexuals	582 (14.5)	236 (6.1)	
Injecting drug users	1,478 (36.7)	625 (16.2)	
Others/unknown	694 (17.2)	47 (1.2)	
Mean CD4 count (SD, range), cells/μL	309.2 (249, 0–2,760)	282 (189, 0–2,217)	<0.001
Late presenter (CD4 ≤200 cells/μL), n/N* (%)	1,269/3,432 (37.0)	1,278/3,749 (34.1)	0.011
Mean plasma HIV RNA load (SD, range), log_10_ copies/mL	4.41 (1.03, 1.7–7.15)	4.73 (0.85, 1.3–7.55)	<0.001
HBsAg-positive, n/N (%)	618/3,805 (16.2)	412/3,776 (10.9)	<0.001
Anti-HCV-positive, n/N (%)	1,288/2,974 (43.4)	707/3,793 (18.6)	<0.001
RPR titer ≥1:8, n/N (%)	255/1,636 (15.6)	400/2,407 (16.6)	0.382

Note: n/N: tests to be positive/tested number

Abbreviations: HBsAg, hepatitis B virus surface antigen; HCV, hepatitis C virus; RPR, rapid plasma reagin; SD: standard deviation

The prevalence of HBV/HIV and HCV/HIV co-infection in the 2004–2007 cohort was 16.2% and 43.4%, respectively, which was significantly higher than that in the 2012–2016 cohort (10.9% and 18.6%, respectively, *P*<0.001). In the 2004–2007 and the 2012–2016 cohort, 15.6% and 16.6% of the patients tested had an RPR titer of 8 or greater, respectively (*P* = 0.382). The number of HIV-positive patients with dual infection with HBV and HCV was 246 out of 3008 patients assessed (8.2%) in the 2004–2007 cohort and 115 out of 3,759 patients (3.1%) in the 2012–2016 cohort (*P*<0.001).

**Fig 1:**
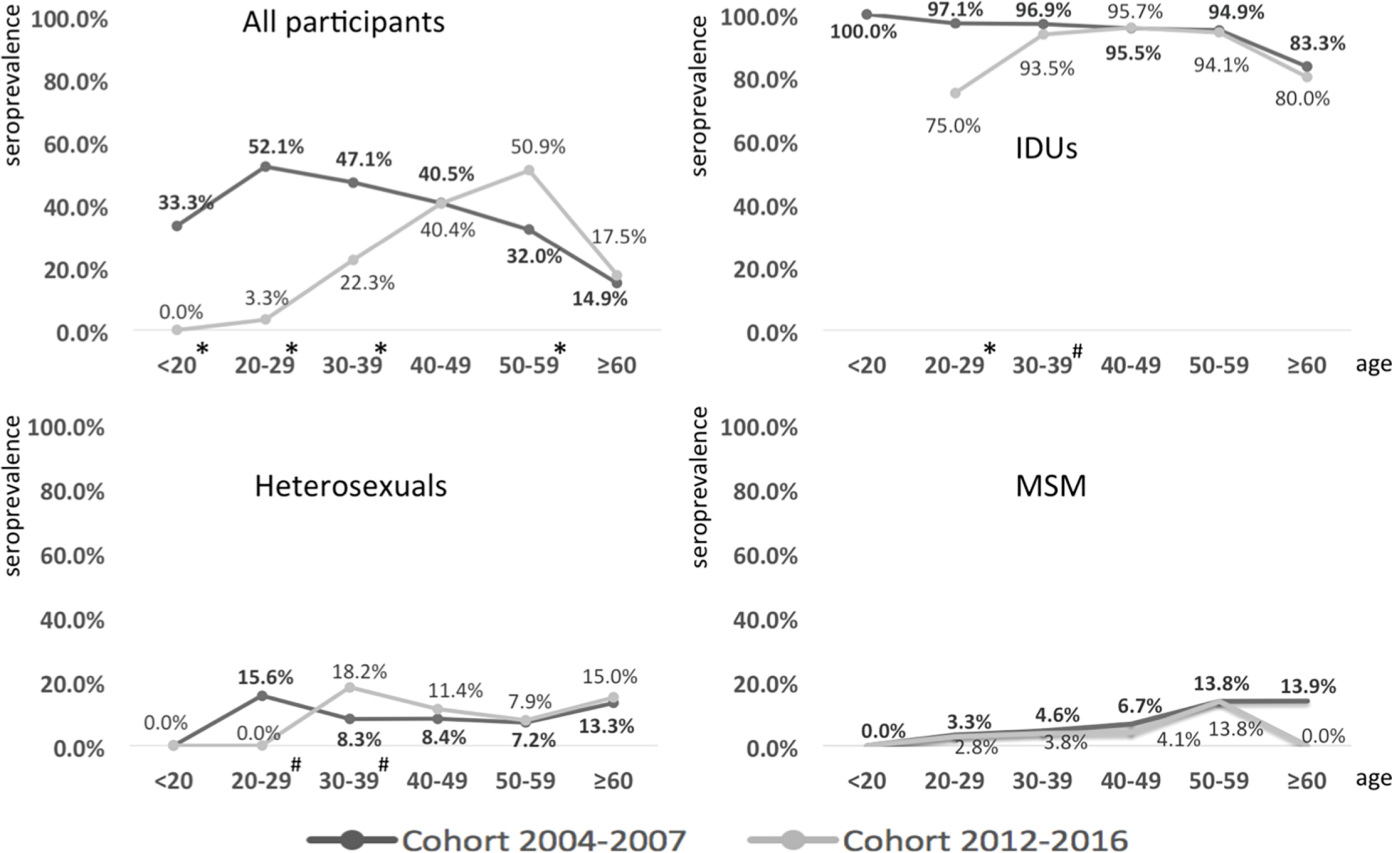
Seroprevalence of hepatitis C virus (HCV) infection among human immunodeficiency virus (HIV)-positive patients of different transmission routes and age groups in the 2004–2007 cohort and 2012–2016 cohort. IDUs: injecting drug users; MSM: men who have sex with men. *P<0.001; ^#^*P*<0.05.

From 2004–2007 to 2012–2016, the overall HCV seroprevalence declined from 96.4% to 94.0% (*P* = 0.02) among IDUs and 5.9% to 3.5% (*P* = 0.002) among MSM; however, no significant change in HCV seroprevalence was observed among heterosexuals (9.4% vs. 10.9%, *P* = 0.59). The HCV seroprevalence in the two cohorts according to age-specific groups including <20 years, 20–29, 30–39, 40–49, 50–59, and ≥60 years are shown in Fig 1. The HCV seroprevalence was significantly lower in the age groups between <20 years to 30–39 years in the 2012–2016 cohort compared to that in the 2004–2007 cohort (*P*<0.001), but was similar among those who were aged ≥40 years between the two cohorts and was higher in the age group of 50–59 years in the 2012–2016 cohort (*P*<0.001), as shown in Fig 1. While the HCV seroprevalence among HIV-positive IDUs remained constantly high across all the age groups in the two cohorts, the overall HCV seroprevalence among MSM increased with age, from 3.3% in the patients aged 20–29 years to 13.9% in those aged ≥60 years in the 2004–2007 cohort, and from 2.8% in the patients aged 20–29 years to 13.8% in those aged 50–59 years in the 2012–2016 cohort (Fig 1). No significant difference was observed in HCV seroprevalence in each age group among MSM between these two cohorts. For the heterosexuals, the overall HCV seroprevalence according to age-specific groups, including 20–29, 30–39, 40–49, 50–59, and ≥60 years, was 15.6%, 8.3%, 8.4%, 7.2%, and 13.3%, respectively, for the 2004–2007 cohort; and the respective seroprevalence was 0%, 18.2%, 11.4%, 7.9%, and 15.0% for the 2012–2016 cohort.

In the 2004–2007 cohort, the patients born between 1970 and 1979 had the highest HCV seroprevalence of 50.6%, while in the 2012–2016 cohort, the highest HCV seroprevalence (44.4%) occurred among the patients born between 1960 and 1969 (Table 2). Among IDUs in the 2004–2007 cohort, the HCV seroprevalence was as high as 96.7% in the patients born between 1970 and 1979, while in the 2012–2016 cohort, the highest HCV seroprevalence (95.1%) was noted among patients born between 1950 and 1959. Among the HIV-positive MSM in the 2004–2007 cohort, the highest HCV seroprevalence was 14.3% in the patients born between 1950 and 1959 and the lowest seroprevalence was 1.4% in those born between 1980 and 1989; in the 2012–2016 cohort, the highest HCV seroprevalence was 14.3% in the patients born between 1950 and 1959, which decreased to 2.6% in those born between 1990 and 1999 (Table 2).

**Table 2:** HCV seroprevalence of HIV-positive patients of different transmission groups and birth years.

Year of birth	Patient number	All patients with available data		Injecting drug users		Men who have sex with men		Heterosexuals	
		2004–2007, n/N	2012–2016, n/N	2004–2007, n/N	2012–2016, n/N	2004–2007, n/N	2012–2016, n/N	2004–2007, n/N	2012–2016, n/N
before 1950	288	32/194 (16.5)	3/21 (14.3)	9/10 (90.0)	1/2 (50.0)	5/43 (11.6)	0/8 (0)	13/97 (13.4)	2/10 (20.0)
1950–1959	551	124/342 (36.3)	44/101 (43.6)	84/87 (96.6)	39/41 (95.1)	14/98 (14.3)	4/28 (14.3)	10/104 (9.6)	1/28 (3.6)
1960–1969	1,565	358/888 (40.3)	167/376 (44.4)	301/313 (96.2)	150/161 (93.2)	16/316 (5.1)	10/159 (6.3)	11/158 (7.0)	6/50 (12.0)
1970–1979	2,492	592/1,171 (50.6)	317/880 (36.0)	500/517 (96.7)	286/301 (95.0)	23/420 (5.5)	22/523 (4.2)	7/86 (8.1)	9/49 (18.4)
1980–1989	2,299	182/379 (48.0)	157/1,737 (9.0)	159/165 (96.4)	100/107 (93.5)	2/140 (1.4)	49/1,545 (3.2)	3/23 (13.0)	7/70 (10.0)
1990–2000	686	0	19/678 (18.6)	0	577/614 (94.0)	0	17/2,907 (2.6)	0	0/23 (0)

n/N: tests to be positive/tested number

Two multivariate analyses to identify the factors associated with HCV seropositivity were conducted among the included patients (Table 3); in model 1, only the patients acquiring HIV through sexual transmission were included, while in model 2, both patients acquiring HIV through sexual transmission and injecting drug use were included. The variables included in both analyses were age, gender, transmission route, baseline CD4 lymphocyte count, baseline PVL, HBsAg, and RPR ≥1:8. In model 1, 2,905 patients with all these variables available were included in the analysis. The adjusted odds ratio (aOR) was 1.03 for per 1-year increase (95% confidence interval [CI] 1.02–1.05). Compared with heterosexuals, the aOR for HCV seropositivity among MSM was 0.47 (95% CI, 0.31–0.72). Among those with RPR test results, the aOR for HCV seropositivity among the patients with an RPR titer ≥1:8 was 1.58 (95% CI, 1.03–2.43). In model 2, 3,289 patients were included for analysis. The aOR for per 1-year increase was similar to that of model 1. Compared with heterosexuals, the aOR for HCV seropositivity among MSM was 0.43 (95% CI, 0.28–0.66). Among those with RPR test results, the aOR for HCV seropositivity among the patients with an RPR titer ≥1:8 was 1.49 (95% CI, 0.99–2.26).

**Table 3:** Multivariate analyses* of associated factors with seropositivity of hepatitis C virus among patients who acquired HIV through sexual transmission and injecting drug use.

Variables	HCV seropositivity					
	Model 1^a^, n = 2,905			Model 2^b^, n = 3,289		
	Adjusted odds ratio	95% confidence interval	*P* value	Adjusted odds ratio	95% confidence interval	*P* value
Age, per 1-year increase	1.03	1.02–1.05	<0.001	1.03	1.02–1.05	<0.001
Transmission route						
Heterosexual contact	Reference			Reference		
Male-to-male contact	0.47	0.31–0.73	0.001	0.43	0.28–0.66	<0.001
Injecting drug use	Not applicable			84.13	50.94–138.96	<0.001
Baseline CD4 count	1.001	1.000–1.001	0.168	1.001	1.000–1.002	0.003
Rapid plasma reagin titer ≥1:8	1.58	1.03–2.43	0.038	1.49	0.99–2.26	0.059

*Variables included were age, gender, transmission route, baseline CD4 number and plasma HIV RNA load, positive hepatitis B surface antigen, and a rapid plasma reagin titer ≥1:8.

^a^ Model 1 only included patients acquiring HIV through sexual transmission for analysis.

^b^ Model 2 included patients acquiring HIV through sexual transmission and injecting drug use for analysis.

When the multivariate analyses were repeated separately in each cohort (S1 Table), we found that the associations between age and transmission route with HCV seropositivity remained similar, but, while CD4 count was significantly associated with HCV seropositivity in the two cohorts, the statistically significant association between an RPR titer≥1:8 and HCV seropositivity was only observed in the 2012–2016 cohort.

## Discussion

The present study demonstrates that the overall HCV seroprevalence has declined among the HIV-positive patients in Taiwan in the post-cART era, though HIV-positive IDUs consistently had the highest HCV seroprevalence across all age groups. On the other hand, the finding of a lower HCV seroprevalence that increased with age among HIV-positive MSM of the later birth cohort were quite similar to that observed in the general population in Taiwan [22]. Traditional factors such as use of contaminated blood products or unsterile equipment or devices in the medical facilities that used to contribute to the previous hyperendemicity of HCV infection among the general population in Taiwan in earlier years were less likely to present for the younger population [5,22–24]. However, sharing needles and diluents continue to be the major risky behavior related to transmission of both HIV and HCV among HIV-positive IDUs [25].

Previous studies have found that the HCV seroprevalence increased along with time despite the cohort effects [5,7]. In the Italian Health Longitudinal Patient Database, HCV seroprevalence increased from 0.24% in 2002 to 0.5% in 2012 [10]. With an 8-fold increase over time, HCV seroprevalence in Czech Republic increased from 0.2% in 2001 to 1.67% in 2015 [26]. However, our study found a decline in the HCV seroprevalence among the HIV-positive patients in the two study cohorts, though the incidence of recent HCV infection from sexually transmitted routes was increasing in recent reports [9,15,27]. The significant decline of HCV seroprevalence in our study is likely attributed to the changing composition of risk groups for HIV transmission between the two cohorts. IDUs had the highest HCV seroprevalence among the HIV-positive patients. In the 2004–2007 cohort, IDUs accounted for 36.7% of the patients included, which decreased to 16.2% in the 2012–2016 cohort; moreover, the patients in the 2012–2016 cohort were significantly younger that those in the 2004–2007 cohort (33.1 *vs*. 38.6 years, *P*<0.001).

Despite of the overall decline of HCV seroprevalence, different patterns of changes of HCV seroprevalence were observed among different risk groups. Compared with IDUs, HCV seroprevalence was much lower among HIV-positive MSM and heterosexuals. With the implementation of harm reduction program to effectively control the HIV outbreak among IDUs between 2003 and 2008, the number of new HIV infections through injecting drug use has decreased dramatically, which only constituted 3.5% of new HIV infections in 2016 [17,28,29]. However, the HCV seroprevalence remains high among the IDUs, with only 2.4% decrease from 96.4% in the 2004–2007 cohort to 94.0% in the 2012–2016 cohort. The findings suggest that sustained harm reduction program is needed to achieve the great impact on reduction of HCV transmission among IDUs in the long-term.

In this study, we found that younger HIV-positive MSM had a lower HCV seroprevalence in our two cohorts, consistent with the trends among the general population [22,23]. Among these sexually transmitted HIV-positive patients, the correlation between syphilis and HCV seropositivity in multivariate analysis suggests that some of the patients in our cohort might acquire HCV infection through sexual transmission [15,16]. Recent studies have shown significantly higher risks of HCV seroconversion among MSM compared to heterosexuals and the association with sexually transmitted infections, traumatic sex, and use of recreational drugs among MSM [30,31]. However, our cross-sectional study shows that HIV-positive MSM had a lower HCV seroprevalence than HIV-positive heterosexuals in our multivariable analysis. The reason for this finding is not clear. However, the misclassification of transmission routes might occur. Some IDUs with HCV infection would report themselves as heterosexuals because of concerns of legal liability. In addition, the finding that HCV seroprevalence increased with age was only noted in MSM, but not heterosexuals, as shown in Fig 1. This phenomenon also suggests a mixture of sexual and IDU transmission in our heterosexual population. Annual follow-up of incident HCV infection is warranted among the at-risk populations because, similar to what has been reported in the developed countries, recent studies have shown that increasing HCV seroconversion among MSM [15,32].

There are several limitations in our study. First, while this was a multicenter study with the patients from several designed hospitals for HIV care around Taiwan in these two cohorts, the participating hospitals were not completely identical in these two cohorts. However, free-of-charge HIV care is provided according to the updated national treatment guidelines and the differences in terms of HCV testing rates between two cohorts would be limited. Second, In the 2004–2007 cohort, any patient who sought HIV care at the participating hospitals was included regardless of antiretroviral treatment experience. The later cohort, instead, only included those HIV-positive patients who were antiretroviral-naïve. Since all of the data from the included patients from different hospitals did not contain personal identifiers, we were not able to exclude the possibility that some of the patients might be included in both cohorts. However, by compared birth date, gender, and transmission route between the two cohorts, we found that less than 10% of patients in both cohorts shared the identical clinical characteristics. Third, the misclassification of risk groups of HIV transmission might confound the findings of HCV seroprevalence among different groups in that heterosexuals or MSM who were IDUs might not identify themselves as IDUs because of concerns about legal liability. Fourth, we used an RPR titer ≥1:8 as a cut-off value for presumed syphilitic infection in the analysis; however, RPR titers might be falsely positive, especially among the IDUs with HCV infection. Therefore, in our multivariate analysis that included only sexually transmitted HIV-positive patients, the results were similar. In the meanwhile, using RPR ≥1:16 and RPR ≥1:32 as cut-off values also yielded the independent association with HCV seropositivity (aOR, 1.83 [95% CI, 1.17–2.85] for RPR ≥1:16 and aOR, 2.20 [95% CI, 1.40–3.47] for RPR ≥1:32) (data not shown).

In conclusion, we found that, in these two cross-sectional studies conducted approximately 10-year apart, HCV seroprevalence among HIV-infected patients decreased with time. HCV infection remained highly prevalent among IDUs among all age groups, but HCV seroprevalence among HIV-infected MSM has declined in the later cohort with an increased number of patients in the young age groups. Co-infection with syphilis was an independent associated factor with HCV seropositivity among sexually transmitted HIV-positive patients.

## Supporting information

S1 Table Multivariate analysis of associated factors with seropositivity of hepatitis C virus (HCV) among patients included in two study cohorts. (PDF)

## References

[1] Perz JF, Armstrong GL, Farrington LA, Hutin YJF, Bell BP. The contributions of hepatitis B virus and hepatitis C virus infections to cirrhosis and primary liver cancer worldwide. J Hepatol. 2006;45: 529–38. doi: 10.1016/j.jhep.2006.05.013 16879891 10.1016/j.jhep.2006.05.013

[2] WHO | Hepatitis C 2017. Available from: http://www.who.int/mediacentre/factsheets/fs164/en/

[3] Gower E, Estes C, Blach S, Razavi-Shearer K, Razavi H. Global epidemiology and genotype distribution of the hepatitis C virus infection. J Hepatol. 2014;61: S45–57. doi: 10.1016/j.jhep.2014.07.027 25086286 10.1016/j.jhep.2014.07.027

[4] Messina JP, Humphreys I, Flaxman A, Brown A, Cooke GS, Pybus OG, et al Global distribution and prevalence of hepatitis C virus genotypes. Hepatol Baltim Md. 2015;61: 77–87. doi: 10.1002/hep.27259 25069599 10.1002/hep.27259 PMC4303918

[5] Mohd Hanafiah K, Groeger J, Flaxman AD, Wiersma ST. Global epidemiology of hepatitis C virus infection: new estimates of age-specific antibody to HCV seroprevalence. Hepatol Baltim Md. 2013;57: 1333–42. doi: 10.1002/hep.26141 23172780 10.1002/hep.26141

[6] Jaeckel E, Cornberg M, Wedemeyer H, Santantonio T, Mayer J, Zankel M, et al Treatment of acute hepatitis C with interferon alfa-2b. N Engl J Med. 2001;345: 1452–57. doi: 10.1056/NEJMoa011232 11794193 10.1056/NEJMoa011232

[7] Hajarizadeh B, Grebely J, Dore GJ. Epidemiology and natural history of HCV infection. Nat Rev Gastroenterol Hepatol. 2013;10: 553–62. doi: 10.1038/nrgastro.2013.107 23817321 10.1038/nrgastro.2013.107

[8] Engle RE, Bukh J, Alter HJ, Emerson SU, Trenbeath JL, Nguyen HT, et al Transfusion-associated hepatitis before the screening of blood for hepatitis risk factors. Transfusion (Paris). 2014;54: 2833–41. doi: 10.1111/trf.12682 24797372 10.1111/trf.12682 PMC5498987

[9] Chan DPC, Sun H-Y, Wong HTH, Lee S-S, Hung C-C. Sexually acquired hepatitis C virus infection: a review. Int J Infect Dis IJID Off Publ Int Soc Infect Dis. 2016;49: 47–58. doi: 10.1016/j.ijid.2016.05.030 27270138 10.1016/j.ijid.2016.05.030

[10] Lapi F, Capogrosso Sansone A, Mantarro S, Simonetti M, Tuccori M, Blandizzi C, et al Hepatitis C virus infection: opportunities for an earlier detection in primary care. Eur J Gastroenterol Hepatol. 2017;29: 271–76. doi: 10.1097/MEG.0000000000000785 27849644 10.1097/MEG.0000000000000785

[11] Sulkowski MS. Viral hepatitis and HIV coinfection. J Hepatol. 2008;48: 353–67. doi: 10.1016/j.jhep.2007.11.009 18155314 10.1016/j.jhep.2007.11.009

[12] Soriano V, Barreiro P, Sherman KE. The changing epidemiology of liver disease in HIV patients. AIDS Rev. 2013;15: 25–31. 23449226

[13] Martinello M, Amin J, Matthews GV, Dore GJ. Prevalence and Disease Burden of HCV Coinfection in HIV Cohorts in the Asia Pacific Region: A Systematic Review and Meta-Analysis. AIDS Rev. 2016;18: 68–80. 27196354

[14] Jin F, Matthews GV, Grulich AE. Sexual transmission of hepatitis C virus among gay and bisexual men: a systematic review. Sex Health. 2016; doi: 10.1071/SH16141 27712618 10.1071/SH16141

[15] Sun H-Y, Chang S-Y, Yang Z-Y, Lu C-L, Wu H, Yeh C-C, et al Recent hepatitis C virus infections in HIV-infected patients in Taiwan: incidence and risk factors. J Clin Microbiol. 2012;50: 781–7. doi: 10.1128/JCM.06014-11 22189113 10.1128/JCM.06014-11 PMC3295121

[16] Dougan S, Evans BG, Elford J. Sexually transmitted infections in Western Europe among HIV-positive men who have sex with men. Sex Transm Dis. 2007;34: 783–90. doi: 10.1097/01.olq.0000260919.34598.5b 17495592 10.1097/01.olq.0000260919.34598.5b

[17] Centers for Disease Control, R.O.C. (Taiwan) [Internet]. Available: http://www.cdc.gov.tw/professional/list.aspx?treeid=3f2310b85436188d&nowtreeid=2285b9745a0a3cbb

[18] Lin T, Chen C-H, Chou P. Effects of combination approach on harm reduction programs: the Taiwan experience. Harm Reduct J. 2016;13: 23 doi: 10.1186/s12954-016-0112-3 27377896 10.1186/s12954-016-0112-3 PMC4932706

[19] Liu J-Y, Lin H-H, Liu Y-C, Lee SS-J, Chen Y-L, Hung C-C, et al Extremely high prevalence and genetic diversity of hepatitis C virus infection among HIV-infected injection drug users in Taiwan. Clin Infect Dis Off Publ Infect Dis Soc Am. 2008;46: 1761–8. doi: 10.1086/587992 18433337 10.1086/587992

[20] Lin K-Y, Cheng C-Y, Li C-W, Yang C-J, Tsai M-S, Liu C-E, et al Trends and outcomes of late initiation of combination antiretroviral therapy driven by late presentation among HIV-positive Taiwanese patients in the era of treatment scale-up. PloS One. 2017;12: e0179870 doi: 10.1371/journal.pone.0179870 28665938 10.1371/journal.pone.0179870 PMC5493332

[21] Sun H-Y, Ko W-C, Tsai J-J, Lee H-C, Liu C-E, Wong W-W, et al Seroprevalence of chronic hepatitis B virus infection among taiwanese human immunodeficiency virus type 1-positive persons in the era of nationwide hepatitis B vaccination. Am J Gastroenterol. 2009;104: 877–84. doi: 10.1038/ajg.2008.159 19259078 10.1038/ajg.2008.159

[22] Chen C-H, Yang P-M, Huang G-T, Lee H-S, Sung J-L, Sheu J-C. Estimation of seroprevalence of hepatitis B virus and hepatitis C virus in Taiwan from a large-scale survey of free hepatitis screening participants. J Formos Med Assoc Taiwan Yi Zhi. 2007;106: 148–55. doi: 10.1016/S0929-6646(09)60231-X 17339159 10.1016/S0929-6646(09)60231-X

[23] Yang J-F, Lin C-I, Huang J-F, Dai C-Y, Lin W-Y, Ho C-K, et al Viral hepatitis infections in southern Taiwan: a multicenter community-based study. Kaohsiung J Med Sci. 2010;26: 461–9. doi: 10.1016/S1607-551X(10)70073-5 20837342 10.1016/S1607-551X(10)70073-5 PMC11916219

[24] Ho MS, Hsu CP, Yuh Y, King CC, Tsai JF, Mau YC, et al High rate of hepatitis C virus infection in an isolated community: persistent hyperendemicity or period-related phenomena? J Med Virol. 1997;52: 370–6. 9260682 10.1002/(sici)1096-9071(199708)52:4<370::aid-jmv4>3.0.co;2-z

[25] Zhang F, Zhu H, Wu Y, Dou Z, Zhang Y, Kleinman N, et al HIV, hepatitis B virus, and hepatitis C virus co-infection in patients in the China National Free Antiretroviral Treatment Program, 2010–12: a retrospective observational cohort study. Lancet Infect Dis. 2014;14: 1065–72. doi: 10.1016/S1473-3099(14)70946-6 25303841 10.1016/S1473-3099(14)70946-6 PMC6051428

[26] Chlibek R, Smetana J, Sosovickova R, Gal P, Dite P, Stepanova V, et al Prevalence of hepatitis C virus in adult population in the Czech Republic—time for birth cohort screening. PloS One. 2017;12: e0175525 doi: 10.1371/journal.pone.0175525 28406947 10.1371/journal.pone.0175525 PMC5391198

[27] van de Laar TJ, Richel O. Emerging viral STIs among HIV-positive men who have sex with men: the era of hepatitis C virus and human papillomavirus. Sex Transm Infect. 2017;93: 368–73. doi: 10.1136/sextrans-2016-052677 27789574 10.1136/sextrans-2016-052677

[28] Lyu S-Y, Su L-W, Chen Y-MA. Effects of education on harm-reduction programmes. Lancet Lond Engl. 2012;379: e28–30. doi: 10.1016/S0140-6736(11)60786-1 10.1016/S0140-6736(11)60786-1 PMC7137150 21851974

[29] Huang Y-F, Yang J-Y, Nelson KE, Kuo H-S, Lew-Ting C-Y, Yang C-H, et al Changes in HIV incidence among people who inject drugs in Taiwan following introduction of a harm reduction program: a study of two cohorts. PLoS Med. 2014;11: e1001625 doi: 10.1371/journal.pmed.1001625 24714449 10.1371/journal.pmed.1001625 PMC3979649

[30] Apers L, Vanden Berghe W, De Wit S, Kabeya K, Callens S, Buyze J, et al Risk factors for HCV acquisition among HIV-positive MSM in Belgium. J Acquir Immune Defic Syndr 1999. 2015;68: 585–93. doi: 10.1097/QAI.0000000000000528 25763786 10.1097/QAI.0000000000000528

[31] Ghisla V, Scherrer AU, Nicca D, Braun DL, Fehr JS. Incidence of hepatitis C in HIV positive and negative men who have sex with men 2000–2016: a systematic review and meta-analysis. Infection. 2017;45: 309–21. doi: 10.1007/s15010-016-0975-y 28005195 10.1007/s15010-016-0975-y

[32] Tsai J-C, Hung C-C, Chang S-Y, Liu W-C, Wu C-H, Su Y-C, et al Increasing incidence of recent hepatitis C virus infection among persons seeking voluntary counselling and testing for HIV and sexually transmitted infections in Taiwan. BMJ Open. 2015;5: e008406 doi: 10.1136/bmjopen-2015-008406 26463221 10.1136/bmjopen-2015-008406 PMC4606383

