# Supplementary material for: Changing seroprevalence of hepatitis C virus infection among HIV-positive patients in Taiwan
Source: PLoS One. 2018 Mar 16;13(3):e0194149. doi: 10.1371/journal.pone.0194149 (PMC5856341; doi:10.1371/journal.pone.0194149)
Supplement: S1 Table — (PDF) [file pone.0194149.s001.pdf]

S1 Table. Multivariate analysis of associated factors with seropositivity of hepatitis C virus (HCV) among patients included in two study cohorts.

Variables	HCV seropositivity					
	2004-2007 cohort, n=1,028			2012-2016 cohort, n=2,261		
	Adjusted odds ratio	95% confidence interval	*P* value	Adjusted odds ratio	95% confidence interval	*P* value
Age, per 1-year increase	1.028	1.007-1.051	0.011	1.030	1.010-1.050	0.007
Transmission route						
Heterosexual contact		Reference			Reference	
Male-to-male contact	0.755	0.407-1.399	0.372	0.285	0.160-0.506	<0.001
Injecting drug use	148.851	64.889-341.455	<0.001	47.063	24.813-89.265	<0.001
Baseline CD4 count	1.001	1.000-1.002	0.018	1.001	1.000-1.002	0.120
Rapid plasma reagin titer ≥1:8	1.046	0.499-2.193	0.905	1.780	1.069-2.965	0.027

Note: The variables included in the analyses were age, gender, transmission route, baseline CD4 lymphocyte count, baseline HIV RNA load, HBsAg, and RPR ≥1:8. Only the patients in each cohort with all these variables available were included in the analysis
